# A 4-week, dose-ranging study comparing the efficacy, safety and tolerability of latanoprost 75, 100 and 125 μg/mL to latanoprost 50 μg/mL (xalatan) in the treatment of primary open-angle glaucoma and ocular hypertension

**DOI:** 10.1186/1471-2415-12-9

**Published:** 2012-05-18

**Authors:** David Eveleth, Carla Starita, Charles Tressler

**Affiliations:** 1 Pfizer Inc, San Diego, California, USA; 2 Pfizer Ltd, Walton-on-the Hill, Tadworth Surrey, UK; 3 Pfizer Inc, New York, New York, USA; 4 Specialty Care Business Unit La Jolla Laboratories Pfizer Inc, 10646 Science Center Drive, San Diego, CA, 92121, USA

## Abstract

**Background:**

Several studies have investigated the effect of latanoprost on intraocular pressure (IOP). We compared the IOP-lowering effects of three higher concentrations of latanoprost with the commercially available concentration of 0.005% (50 μg/mL) in patients with primary open-angle glaucoma or ocular hypertension.

**Methods:**

Treatment-naive subjects or those receiving IOP-lowering medication with baseline IOP levels of ≥24 mmHg and ≤36 mmHg in at least one eye after washout were randomized to receive an evening dose of latanoprost 50, 75, 100, or 125 μg/mL for 4 weeks. At weeks 1, 2, 3, and 4, ocular examinations were performed and IOP was measured. Ocular symptoms and adverse events were monitored. The primary efficacy endpoint was the change in IOP from baseline to week 4 at 8 a.m. and 4 p.m. for the per protocol (PP) population using a "worse eye" analysis. Secondary efficacy endpoints were change in IOP at each time point from baseline across all visits, and percentage change in IOP from baseline to week 4 at 8 a.m.

**Results:**

In all, 282 patients were randomized and treated; 274 were included in the PP population. Treatment groups were similar at baseline; 68% were diagnosed with primary open-angle glaucoma. Mean baseline IOP levels were comparable across treatments. There were no statistically significant differences in IOP reductions from baseline to week 4 at either time point between those treated with higher concentrations of latanoprost versus those receiving 50 μg/mL. Least squares mean IOP changes at 8 a.m. were −10.13, -9.59, -10.02, and −9.06 mmHg for latanoprost 50, 75, 100, and 125 μg/mL, respectively, and at 4 p.m. were −8.90, -8.29, -8.81, and −8.34 mmHg, respectively. Results of secondary efficacy analyses supported those of the primary analysis. Conjunctival hyperemia, the most commonly reported adverse event, occurred in 16.9%, 18.6%, 20.8% and 15.9% of subjects receiving latanoprost 50, 75, 100, and 125 μg/mL, respectively.

**Conclusions:**

IOP reductions were observed in all treatment groups postbaseline, with no clinically relevant or statistically significant differences detected favoring any of the higher concentrations of latanoprost compared with latanoprost 50 μg/mL. All doses of latanoprost were well tolerated.

**Trial registration:**

Clinical Trials.gov Identifier NCT01379144.

## Background

Primary open-angle glaucoma (POAG) is a progressive optic neuropathy with characteristic changes in the optic nerve head, including optic nerve cupping and thinning of the neuroretinal rim [1,2], which are associated with loss of peripheral vision [2]. In patients with POAG or ocular hypertension, disease progression can often be delayed or even halted by lowering the intraocular pressure (IOP) by surgical [3] or medical [4,5] treatment. Latanoprost is a prostaglandin F_2α_ (PGF_2α_) analog that, along with other agents of its class, has been shown to increase uveoscleral outflow in patients with glaucoma [6] and ocular hypertension [7], and in healthy subjects [8]. These effects were not found to be accompanied by any reduction in aqueous humor production or dynamics [6,8,9]. The mechanism for the increase in uveoscleral outflow has not been completely elucidated, but there is evidence suggesting that it may involve upregulation of matrix metalloproteinases and remodeling of the extracellular matrix of the ciliary muscle and/or sclera [10,11].

There are relatively few studies in which the effect of latanoprost dose on IOP has been investigated [12]. One early study showed that latanoprost resulted in a dose-dependent reduction in IOP at 8 h (3.4, 4.9, and 5.9 mmHg for 25, 50, and 100 μg/mL, respectively) [13]. A second small exploratory study showed a dose-dependent effect on day 2 but no difference on day 28 for 35, 60, or 115 μg/mL [14]. There were no differences between groups in investigator-evaluated hyperemia scores, but patients receiving the highest concentration reported more ocular irritation (6/15 in 115 μg/mL group vs. 2/15, 1/15, and 3/15 in the placebo, 35 μg/mL, and 60 μg/mL groups, respectively) [14]. Other studies evaluating multiple dosing effects concluded that once-daily dosing with 0.005%/0.006% latanoprost concentrations was similar to or more effective than twice-daily dosing at concentrations ranging from 0.005% to 0.001% in healthy volunteers and in patients with ocular hypertension and/or glaucoma [15-21].

Yet, few of these studies had sufficient numbers of patients/subjects for statistical comparisons between groups. In the second Alm study [14], the maximum diurnal IOP reductions observed were on the order of 5 mm Hg, and there was a trend (but not a statistical conclusion) towards lower IOP at higher doses at the end of the study. Given the size of the study (15 pts/arm) and the variance assumptions this study had only an 80% power to detect a difference between arms of 2.8 mm Hg (alpha = 0.05% one sided). The possibility therefore exists that there is a dose-dependent increase in IOP lowering activity at doses beyond 50ug/ml large enough to be clinically significant. We present results of a dose-ranging study evaluating the efficacy, safety and tolerability of the marketed concentration of latanoprost 0.005% (50 μg/mL; Xalatan) compared to 75, 100, and 125 μg/mL in subjects with POAG and ocular hypertension.

## Methods

### Study design

This was a 4-week multicenter, randomized, double-masked, dose-ranging, parallel-group study comparing three higher concentrations of latanoprost (75, 100, and 125 μg/mL) with latanoprost 50 μg/mL. This study was conducted in accordance with the International Conference on Harmonization Guideline for Good Clinical Practice and the Declaration of Helsinki and the protocol approved by the independent ethics committees/institutional review boards responsible for each participating institution. Written informed consent was obtained from all subjects prior to enrollment in the study.

Ethics committees/IRBS included Ministerio da Saude Hospitais Civis de Lisboa, Lisbon, Portugal; Ethics Committee of Institute of Aviation Medicine, Prague, Czech Republic; South East Sydney Area Health Research Ethics Committee, Sydney, Australia; Comite Consultatif de Protection des Personnes dans la Recherche Biomedicale, Hopital Tarnier, Paris, France; AIBILI Associacao para Investigacao Biomedica e Inovacao em Luz e Imagem Azinhaga de Santa Coma, Coimbra, Portugal; Solihull Local Research Ethics Committee, Birmingham, UK; East Birmingham Local Research Ethics Committee Birmingham Heartlands Hospital, Birmingham, UK; AKADIMOS Ophthalmology Centre of Northern Greece, Thessaloniki, Greece; Ethics Committee of OSL, Prague, Czech Republic; Aga Khan University Hospital IRB, Karachi, Pakistan; Unidada Local de Saude de Matosinhos, Matosinhos, Portugal; Research Ethics Committee Glasgow Royal Infirmary, Glasgow, Scotland, UK; Sunderland Local Research Ethics Committee, Sunderland, UK; The Layton Rahmatulla Benevolent Trust Free Eye & Cancer Hospital, Lahore, Pakistan; The Ethics Committee of the Faculty of Medicine Cholalongkom University, Bangkok, Thailand; The Ethical Review Committee on Research Involving Human Subjects, Faculty of Medicine, Siriraj Hospital, Mahidol University, Bangkok, Thailand; eticka komise of the General Faculty Hospital, Charles University, Prague, Czech Republic; Royal Adelaide Hospital Research Ethics Committee, Adelaide, Australia; Medical Ethics Committee, Dow Medical College and Civil Hospital, Karachi, Pakistan; University Hospital Brno, Brno, Czech Republic; PGMI/Service Hospital Ethics Committee, Lahore, Pakistan.

### Subjects

Male or female subjects ≥18 years of age with POAG or ocular hypertension requiring unilateral or bilateral administration of IOP-lowering treatment, including subjects who were naïve to such treatment, were enrolled. It was recommended, but not required, that subjects have an IOP ≥17 mmHg and ≤36 mm in at least one eye at screening to be eligible. After a washout period in subjects on prior medications (see “Treatment and assessments” below) subjects were reassessed for eligibility and required to have an IOP ≥24 mmHg and ≤36 mmHg in at least one eye at 8 a.m. at the baseline visit. Exclusion criteria included: closed/barely open anterior chamber angle or a history of acute angle closure; history of discontinued prostaglandin IOP-lowering treatment, unless the reason for discontinuation was participation in a clinical study; ocular surgery or argon laser trabeculoplasty in one or both eyes within 3 months prior to the screening visit; current use of contact lenses; use or anticipated requirement during the study period of topical medication known to affect IOP; anticipated need to modify systemic medication known to affect IOP during the study period; any condition in one or both eyes that would prevent reliable applanation tonometry; ocular inflammation/infection in one or both eyes within 1 month prior to the screening visit or during the washout period; known hypersensitivity to benzalkonium chloride or to any other component of the study medication; other abnormal ocular conditions or symptoms that prevented the subject from participating in the study, according to the investigator’s judgment; participation in any other ocular or systemic clinical study within 1 month prior to the screening visit; other severe, acute, or chronic medical or psychiatric condition or laboratory abnormality that, in the investigator’s judgment, could have increased the risk associated with study participation or study drug administration, or may have interfered with the interpretation of the study results; uncontrolled systemic disease; women of childbearing potential who were not using adequate contraceptive methods or who were pregnant or nursing.

### Treatments and assessments

Subjects were assessed for eligibility at a screening visit that took place up to 4 weeks prior to the baseline visit. At screening, ocular and medical histories and previous and concomitant medications were documented; ophthalmoscopy, refraction, biomicroscopy, and a slit lamp examination (to evaluate hyperemia) were performed; and best-corrected visual acuity (BCVA) and IOP were measured. Conjunctival hyperemia was graded according to a 4-point photographic reference scale ranging from 0 (none) to 3 (severe). Throughout the study, IOP was measured in both eyes (the right eye preceding the left eye) using a calibrated Goldmann applanation tonometer. The measurement was done by two study personnel (an operator and a recorder) as recommended by the guidelines from the Eye Care Technology Forum [22]. Measurements were repeated in the same eye twice consecutively. If the difference in measurements was ≤2 mmHg, the mean of these measurements was recorded. If the difference in measurements was >2 mmHg, a third measurement was made and the median of the three measurements was recorded. IOP was measured after other ophthalmic examinations but prior to pupil dilation.

Subjects who were receiving IOP-lowering treatment and who met the eligibility criteria entered into a washout period, the duration of which depended on their current IOP treatment (4 weeks for prostaglandin analogs; 3 weeks for beta-blockers [alone or in combination] or oral carbonic anhydrase inhibitors; 2 weeks for epinephrine and dipivefrin or adrenergic agonists; and 1 week for topical carbonic anhydrous inhibitors, pilocarpine, carbachol and aceclidine). Subjects could attend the study site for safety monitoring during the washout period at the discretion of the investigator and/or the subject’s choice. For subjects not receiving IOP-lowering medications, the screening visit could be combined with the baseline visit.

At the baseline visit, examinations performed at screening were repeated except for refraction; ocular symptoms and adverse events were assessed; and iris/en face photographs were taken (2 sets for each eye; one retained at the study center and one sent to a central reading center). Baseline IOP was measured at 8 a.m. and 4 p.m. (± 1 h). Subjects were randomized to receive latanoprost 50, 75, 100, or 125 μg/mL in a 1:1:1:1 ratio with a computerized randomization list using the random permuted blocks method. A multiple of the block size (n = 8) was distributed to each study center, where the investigator assigned subject numbers consecutively. Subjects were instructed to instill one drop of study medication in one or both eyes in the evening of the baseline visit between 7 p.m. and 9 p.m. and every evening during the entire treatment period with the last dose administered in the evening of the day preceding the week 4 visit. All concentrations of latanoprost contained the same concentration of benzalkonium chloride (0.2 mg/ml).

Follow-up visits occurred at weeks 1, 2, 3, and 4. At each visit, biomicroscopy was performed; BCVA, conjunctival hyperemia, and IOP were evaluated; and ocular symptoms, adverse events, and concomitant medications were recorded. At least 12 h must have elapsed between administration of the evening dose of study medication and measurement of the morning IOP. At week 4, ophthalmoscopy was performed and iris/en face photographs were repeated. Subjects were contacted by telephone 1 week after the week 4 visit to elicit additional information concerning adverse events.

Adverse events, both observed and volunteered, were monitored by investigators throughout the study. The severity of events (mild, moderate, or severe) and the investigator’s opinion concerning whether the event was related to study drug were noted. Serious adverse events were those that were life-threatening, required inpatient hospitalization/prolongation of hospitalization, caused persistent or significant disability/incapacity, or resulted in congenital anomaly/birth defect or death. Adverse events were followed until they resolved or stabilized. Adverse events were considered treatment-emergent if they occurred between baseline and week 4 or if they were present at baseline but increased in severity and/or seriousness during the study.

### Endpoints and analyses

Efficacy analyses were performed for both the study eye and the worse eye. The study eye was defined as any eye meeting all inclusion and no exclusion criteria; if both eyes were eligible, the mean value of the IOP recording in both eyes at a given time point was included in analyses. The worse eye was the study eye with the highest baseline 8 a.m. IOP; data from the right eye were used in subjects with two study eyes with equal 8 a.m. IOP levels at baseline. The intention-to-treat (ITT) population included all subjects who received at least one dose of study medication; separate ITT analyses were performed using observed data and using the last observation carried forward (LOCF) method to impute missing IOP data. The per-protocol (PP) population included all subjects who completed the study treatment period without major protocol violations.

The primary efficacy endpoint was the change in IOP from baseline to week 4 (day 28; 8 a.m. and 4 p.m.) in the worse eye, and the primary analysis of this endpoint was conducted in the PP population. Pairwise comparisons of latanoprost 75 μg/mL, 100 μg/mL and 125 μg/mL versus latanoprost 50 μg/mL were made using analysis of covariance (ANCOVA) with baseline IOP as the covariate and center and dose group as factors. A point estimate (least square mean), p-value and a two-sided 90% confidence interval (where the upper limit corresponded to the one-sided test of 5%) for the adjusted treatment differences were calculated. If the treatment effect was not significant, no further models were fitted. If the treatment effect was significant, the model was refitted with terms for treatment by covariate interactions to assess the robustness of the treatment effect across covariates. Standardized residuals were plotted for all models fitted to validate model assumptions. To test the robustness of the results of the primary analysis, analyses were repeated in the PP population for study eyes and in ITT population for both worse and study eyes.

For both worse and study eyes, secondary efficacy endpoints included IOP change from baseline to weeks 1, 2, and 3, analyzed using the PP population, and percentage change in IOP at 8 a.m. 4 weeks postbaseline, analyzed using both the PP and the ITT populations. The statistical significance of pairwise comparisons between latanoprost 50 μg/mL and each higher concentration was tested at each visit and time point using the same model as for the primary efficacy analysis. These efficacy comparisons were one-sided and performed at the 5% level of significance; no adjustment for multiple comparisons was made. The influence of potential prognostic factors (ie, age, gender) on the change in IOP was evaluated using frequency tables for the PP population on the worse eye.

Safety analyses were based on both eyes using the ITT population. Adverse events were classified by system organ class and preferred term using the Medical Dictionary for Regulatory Activities (MedDRA) version 2.3, and treatment-emergent adverse events were tabulated by treatment group. The frequency of cells/flare in the anterior chamber and ocular symptoms was tabulated as was the change from baseline in iris pigmentation and eyelash growth. Hyperemia was assessed using the same scale that was used in the phase III studies of latanoprost (25,28), with 0 = None (no hyperemia), 1 = Mild (reddening of the palpebral or bulbar conjunctiva). 2 = Moderate (bright reddening of palpebral or bulbar conjunctiva) and 3 = Severe (deep, bright, and diffuse reddening of palpebral or bulbar conjunctiva).

The sample size of 240 subjects was based on clinical judgment rather than on formal power calculations. Assuming a standard deviation of 4 mmHg, a study recruiting 50 evaluable subjects per treatment arm would provide 80% power to detect a difference of 2.0 mmHg in the change from baseline IOP (pairwise comparison of higher latanoprost concentrations vs latanoprost 50 μg/mL) at 28 days at the 5% level of significance (one-sided test). Furthermore, the corresponding power would be 58% to detect a difference of 1.5 mmHg.

## Results

**Figure 1:**
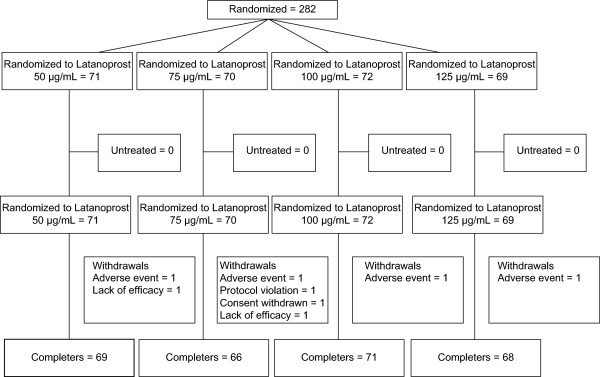
**Subject disposition.** Flow chart of subjects detailing number of randomized subjects in each group, number of subjects withdrawing from the study, and the PP population by group.

Between January and March 2003, a total of 282 subjects were randomized at 25 centers (Australia, 3; Czech Republic, 5; France, 4; Greece, 1; Pakistan, 4; Portugal, 3; Thailand, 2; United Kingdom, 3) and were included in the ITT population (Figure 1). At least 94% of subjects in each treatment group completed the study. The mean duration of study treatment in the ITT population was between 27 and 28 days in each dose group (range, 4 to 31 days). Eight subjects withdrew from the study, two due to lack of efficacy, one withdrew consent, one due to a protocol violation (noncompliance), and four due to adverse events (1 in each treatment group; 2 with eye irritation and 2 with conjunctival vascular disorder, all considered to be study drug related). The PP population included 274 subjects (50 μg/mL, n = 69; 75 μg/mL, n = 66; 100 μg/mL, n = 71; 125 μg/mL, n = 68). The PP population and the ITT observed cases populations are identical.

Treatment groups were similar with respect to demographic and baseline characteristics (Table 1). In the overall population, the mean age was 60.2 years, ranging from 20 to 89 years. Overall proportions of males and females were similar (male, 51%; female, 49%), and 79% of subjects were Caucasian. Most subjects (68%) were diagnosed with primary open-angle glaucoma in both eyes while approximately 25% had a diagnosis of ocular hypertension in both eyes. At screening, the most commonly used IOP-lowering medications in each treatment group were beta-blockers and prostaglandins, used by approximately 40% and 30% of subjects, respectively. In the PP population, mean baseline IOP levels were comparable across treatments at 8 a.m. and 4 p.m. in both worse and study eyes.

**Table 1:** Demographics and baseline characteristics

**Latanoprost concentration (μg/mL)**				
	**50**	**75**	**100**	**125**
**ITT Population, N**	71	70	72	69
**Sex, n (%)**				
Male	32 (45.1)	35 (50.0)	42 (58.3)	35 (50.7)
Female	39 (54.9)	35 (50.0)	30 (41.7)	34 (49.3)
**Race, n (%)**				
Caucasian	56 (78.9)	57 (81.4)	55 (76.4)	56 (81.2)
Black	1 (1.4)	0 (0)	2 (2.8)	1 (1.4)
Asian	14 (19.7)	13 (18.6)	15 (20.8)	12 (17.4)
**Age (years)**				
Mean ± SD	59.5 ± 12.8	59.5 ± 12.0	60.6 ± 13.8	61.2 ± 12.7
Median	61.0	59.0	60.5	63.0
Range	20-82	26-85	20-85	26-89
**Study eye, n (%)**				
Right	16 (22.5)	15 (21.4)	13 (18.1)	11 (15.9)
Left	8 (11.3)	8 (11.4)	11 (15.3)	16 (23.2)
Both	47 (66.2)	47 (67.1)	48 (66.7)	42 (60.9)
**Treated eye, n (%)**				
Right	7 (9.9)	5 (7.1)	3 (4.2)	4 (5.8)
Left	1 (1.4)	1 (1.4)	2 (2.8)	4 (5.8)
Both	63 (88.7)	64 (91.4)	67 (93.1)	61 (88.4)
**Worse eye, n (%)**				
Right	50 (70.4)	43 (61.4)	39 (54.2)	36 (52.2)
Left	21 (29.6)	27 (38.6)	33 (45.8)	33 (47.8)
**Eye color, n (%)**				
Homogenous blue, gray or green	8 (11.3)	11 (15.7)	4 (5.6)	11 (15.9)
Homogenous brown	45 (63.4)	43 (61.4)	46 (63.9)	40 (58.0)
Blue-brown or gray-brown	10 (14.1)	12 (17.1)	11 (15.3)	11 (15.9)
Green-brown	4 (5.6)	3 (4.3)	8 (11.1)	4 (5.8)
Yellow-brown	3 (4.2)	1(1.4)	3 (4.2)	3 (4.3)
Different*	1 (1.4)	0 (0.0)	0 (0.0)	0 (0.0)
**Diagnosis**^**†**^**, n (%)**				
Primary open-angle glaucoma	53 (74.6)	44 (62.9)	47 (65.3)	47 (68.1)
Ocular hypertension	16 (22.5)	18 (25.7)	21 (29.2)	16 (23.2)
Different*	2 (2.8)	8 (11.4)	4 (5.6)	6 (8.7)
**IOP-lowering medication at**				
**screening, n%**				
Alpha agonists	3 (4.2)	5 (7.1)	6 (8.3)	1 (1.4)
Beta-blockers	33 (46.5)	28 (40.0)	26 (36.1)	23 (33.3)
Carbonic anhydrase inhibitors	7 (9.9)	8 (11.4)	2 (2.8)	2 (2.9)
Combination drugs	9 (12.7)	6 (8.6)	4 (5.6)	5 (7.2)
Parasympathomimetics	0 (0.0)	1 (1.4)	0 (0.0)	2 (2.9)
Prostaglandins	23 (32.4)	19 (27.1)	20 (27.8)	20 (29.0)
Sympathomimetics	1 (1.4)	1 (1.4)	0 (0.0)	0 (0.0)
**PP Population, N**	69	66	71	68
**Baseline IOP**				
**Worse eye** **8 a.m.**				
Mean ± SD	26.9 ± 3.1	26.6 ± 3.0	26.5 ± 2.5	26.8 ± 2.8
Range	24.0 – 36.0	24.0 – 36.0	24.0 – 35.0	24.0 – 36.0
**4 p.m.**				
Mean ± SD	25.3 ± 3.6	24.6 ± 3.5	25.3 ± 3.1	25.7 ± 3.7
Range	16.5 – 37.0	15.0 –35.0	17.5 – 34.0	18.0 – 36.0
**Study eye**				
**8 a.m**.				
Mean ± SD	26.5 ± 2.8	26.1 ± 2.5	26.2 ± 2.4	26.4 ± 2.4
Range	24.0 – 34.0	24.0 – 36.0	24.0 – 35.0	24.0 – 34.0
**4 p.m.**				
Mean ± SD	25.1 ± 3.4	24.3 ± 3.1	25.1 ± 2.9	25.3 ± 3.3
Range	16.5 – 35.0	15.5 – 34.0	18.0 – 34.0	18.0 – 36.0

*IOP* intraocular pressure, *ITT* intention-to-treat, *PP* per protocol, *SD* standard deviation.

*If the diagnosis/eye color of a subjects eye was not the same in both eyes, the outcome was given as different.

^**†**^By subject (i.e. diagnoses for both eyes of the subject were combined).

### Efficacy

At week 4, least square mean IOP reductions from baseline in the worse eye in the PP population occurred in all dose groups at both 8 a.m. and 4 p.m. with somewhat greater reductions at 8 a.m. (between 9 and 10 mmHg) than at 4 p.m. (between 8 and 9 mmHg; Table 2). None of the pairwise differences between the latanoprost 50 μg/mL dose group and the 75, 100, or 125 μg/mL groups was statistically significant (primary efficacy endpoint). These findings were confirmed by results of analyses in the ITT population using LOCF (Table 3). An analysis of the impact of baseline prognostic factors on change in IOP in the worse eye found no differences between the high concentration groups and the latanoprost 50 μg/mL group at week 4 based on age or gender. For the study eye, no significant differences were noted between those treated with latanoprost 50 μg/mL and subjects in the higher concentration groups in least square mean IOP change from baseline to week 4 at either 8 a.m. or 4 p.m. (data not shown).

**Table 2:** Change in IOP (mmHg) from baseline to week 4 (worse eye, ANCOVA, PP population)

	**Latanoprost concentration (μg/mL)**			
	**50**	**75**	**100**	**125**
N	69	66	71	68
**8 a.m. IOP**				
LS mean change (mmHg)*	−10.13	−9.59	−10.02	−9.06
Adjusted difference in LS mean ± SEM^**†**^		0.54 ± 0.46	0.12 ± 0.45	1.07 ± 0.45
(90% CI)		(−0.22, 1.29)	(−0.63, 0.86)	(0.32, 1.82)
p-value^**‡**^		0.879	0.602	0.990
**4 p.m. IOP**				
LS mean change (mmHg)*	−8.90	−8.29	−8.81	−8.34
Adjusted difference in LS mean ± SEM^**†**^		0.60 ± 0.47	0.09 ± 0.45	0.55 ± 0.46
(90% CI)		(−0.16, 1.37)	(−0.67, 0.84)	(−0.20, 1.31)
p-value^**‡**^		0.902	0.575	0.885

*ANCOVA* analysis of covariance, *CI* confidence interval, *IOP* intraocular pressure, *LS* least square, *PP* per protocol, *SEM* standard error of the mean.

*Adjusting for baseline IOP (covariate) with dose group and center as factors.

^**†**^Higher concentration minus latanoprost 50 μg/mL; a negative difference in LS mean indicates that the higher concentration has a greater effect than latanoprost 50 μg/mL.

^**‡**^One-sided p-value.

**Table 3:** Change in IOP (mmHg) from baseline to week 4 (worse eye, ANCOVA, ITT population using LOCF)

**Latanoprost concentration (μg/mL)**				
	**50**	**75**	**100**	**125**
N	71	70	72	69
**8 a.m. IOP**				
LS mean change (mmHg)*	−9.72	−9.49	−10.04	−9.23
Adjusted difference in LS mean ± SEM^**†**^		0.23 ± 0.58	−0.32 ± 0.58	0.49 ± 0.58
(90% CI)		(−0.72, 1.19)	(−1.27, 0.63)	(−0.47, 1.45)
p-value^**‡**^		0.657	0.289	0.802
**4 p.m. IOP**				
LS mean change (mmHg)*	−8.47	−7.96	−8.84	−8.49
Adjusted difference in LS mean ± SEM^**†**^		0.51 ± 0.63	−0.36 ± 0.62	−0.02 ± 0.63
(90% CI)		(−0.53, 1.55)	(−1.39, 0.67)	(−1.06, 1.02)
p-value^**‡**^		0.792	0.280	0.488

*ANCOVA* analysis of covariance, *CI* confidence interval, *IOP* intraocular pressure, *LS* least square, *ITT* intent to treat, *SEM* standard error of the mean.

*Adjusting for baseline IOP (covariate) with dose group and center as factors.

^**†**^Higher concentration minus latanoprost 50 μg/mL; a negative difference in LS mean indicates that the higher concentration has a greater effect than latanoprost 50 μg/mL.

^**‡**^One-sided p-value.

**Figure 2:**
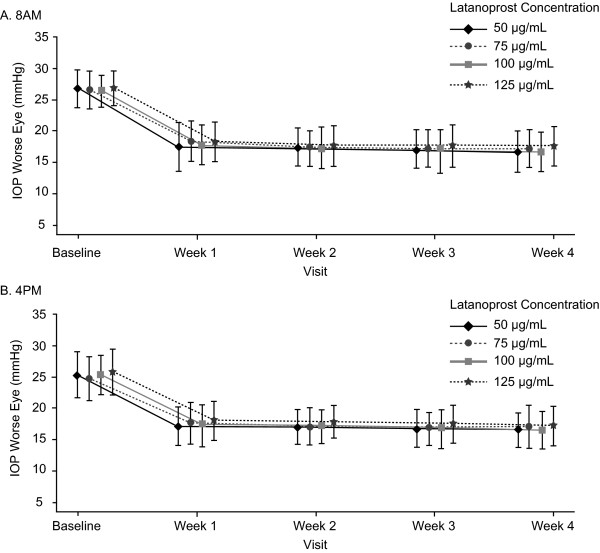
**Mean IOP at each time point in the study.** Mean IOP (mmHg) during treatment at **A**) 8 a.m. and **B**) 4 p.m. for worse eye (PP population). Points indicate mean IOP (mm Hg) for each group at each time point. Error bars +/− SD. Each group is slightly offset in the graph for clarity.

The greatest mean IOP reductions in the worse eye were observed between baseline and week 1, with only slight additional reductions at weeks 2, 3, and 4 (Figure 2A and B). As at week 4, least square mean IOP reductions at weeks 1 through 3 were substantial (>8 mmHg) at each time point and were somewhat greater at 8 a.m. than at 4 p.m. (Table 4). There were no significant differences between latanoprost 50 μg/mL and the higher concentrations of latanoprost in least square mean IOP reductions at any time point during the first three weeks of treatment (Table 4).

**Table 4:** Change in IOP (mmHg) from baseline at weeks 1 to 3 (worse eye, ANCOVA, PP population)

	**Latanoprost concentration (μg/mL)**			
	**50**	**75**	**100**	**125**
**Week 1**				
**8 a.m. IOP**				
N	69	66	71	68
LS mean change (mmHg)*	−9.11	−8.38	−8.85	−8.40
Adjusted difference in LS mean ± SEM^**†**^		0.73 ± 0.50	0.25 ± 0.50	0.71 ± 0.50
(90% CI)		(−0.10, 1.57)	(−0.57, 1.07)	(−0.11, 1.54)
p-value^**‡**^		0.926	0.696	0.922
**4 p.m. IOP**				
N	69	66	71	68
LS mean change (mmHg)*	−8.07	−7.63	−8.04	−7.46
Adjusted difference in LS mean ± SEM^**†**^		0.44 ± 0.48	0.03 ± 0.47	0.61 ± 0.47
(90% CI)		(−0.35, 1.22)	(−0.74, 0.79)	(−0.17, 1.38)
p-value^**‡**^		0.820	0.522	0.902
**Week 2**				
**8 a.m. IOP**				
N	69	66	71	68
LS mean change (mmHg)*	−9.29	−9.56	−9.31	−9.05
Adjusted difference in LS mean ± SEM^**†**^		−0.27 ± 0.44	−0.02 ± 0.43	0.24 ± 0.44
(90% CI)		(−1.00, 0.46)	(−0.73, 0.70)	(−0.49, 0.96)
p-value^**‡**^		0.269	0.485	0.705
**4 p.m. IOP**				
N	69	66	71	67
LS mean change (mmHg)*	−8.28	−8.19	−8.21	−7.65
Adjusted difference in LS mean ± SEM^**†**^		0.08 ± 0.41	0.07 ± 0.40	0.63 ± 0.41
(90% CI)		(−0.60, 0.76)	(−0.60, 0.73)	(−0.04, 1.30)
p-value^**‡**^		0.578	0.565	0.939
**Week 3**				
**8 a.m. IOP**				
N	68	65	71	66
LS mean change (mmHg)*	−9.68	−9.72	−9.79	−9.15
Adjusted difference in LS mean ± SEM^**†**^		−0.04 ± 0.46	−0.12 ± 0.45	0.53 ± 0.45
(90% CI)		(−0.79, 0.71)	(−0.85, 0.62)	(−0.22, 1.28)
p-value^**‡**^		0.463	0.396	0.879
**4 p.m. IOP**				
N	68	64	71	66
LS mean change (mmHg)*	−8.61	−8.60	−8.65	−8.06
Adjusted difference in LS mean ± SEM^**†**^		0.01 ± 0.45	−0.04 ± 0.44	−0.55 ± 0.45
(90% CI)		(−0.73, 0.76)	(−0.78, 0.68)	(−0.18, 1.29)
p-value^**‡**^		0.512	0.464	0.892

*ANCOVA* analysis of covariance, *CI* confidence interval, *IOP* intraocular pressure, *LS* least square, *PP* per protocol, *SEM* standard error of the mean.

*Adjusting for baseline IOP (covariate) with dose group and center as factors.

^**†**^Higher concentration minus latanoprost 50 μg/mL; a negative difference in LS mean indicates that the higher concentration has a greater effect than latanoprost 50 μg/mL.

^**‡**^ One-sided p-value.

Least square mean percentage IOP changes from baseline to week 4 at 8 a.m. ranged from 33.3% to 37.3% across worse and study eyes (Table 5). Differences between latanoprost 50 μg/mL and the higher concentration groups in percentage IOP changes were not statistically significant for either the worse or study eye.

**Table 5:** Percentage change in IOP (mmHg) from baseline to week 4 (ANCOVA, PP population)

	**Latanoprost concentration (μg/mL)**			
	**50**	**75**	**100**	**125**
N	69	66	71	68
**Worse eye**				
**8 a.m. IOP**				
LS mean change (mmHg)*	−37.6	−35.5	−37.3	−33.5
Difference in LS mean change ± SEM^**‡**^		2.2 ± 1.7	0.3 ± 1.7	4.1 ± 1.7
(90% CI)		(−0.06, 5.0)	(−2.4, 3.1)	(1.3, 6.8)
p-value^**‡**^		0.899	0.581	0.992
**Study eye**				
**8 a.m. IOP**				
LS mean change (mmHg)*	−37.2	−35.0	−36.4	−33.3
Difference in LS mean change ± SEM^**‡**^		2.2 ± 1.7	0.8 ± 1.6	3.8 ± 1.6
(90% CI)		(−0.6, 4.9)	(−1.9, 3.4)	(1.1, 6.5)
p-value^**‡**^		0.906	0.682	0.990

*ANCOVA* analysis of covariance, *CI* confidence interval, *IOP* intraocular pressure, *LS* least square, *PP* per protocol, *SEM* standard error of the mean.

*Adjusting for baseline IOP (covariate) with dose group and center as factors.

^**‡**^Higher concentration minus latanoprost 50 μg/mL; a negative difference in LS mean indicates that the higher concentration has a greater effect than latanoprost 50 μg/mL.

^**‡**^One-sided p-value.

### Safety

During treatment, 38% (27/71), 47% (33/70), 54% (39/72) and 45% (31/69) of subjects in the latanoprost 50, 75, 100 and 125 μg/mL groups, respectively, reported at least one treatment-emergent adverse event, the majority of which were eye disorders. Only one subject had a serious adverse event (severe endocarditis after 15 days of treatment, not considered treatment-related). Adverse events reported by ≥2% of subjects in any treatment group are shown in Table 6. The most commonly reported adverse event in all treatment groups was conjunctival hyperemia, which was somewhat more frequent in the latanoprost 100 μg/mL group than in the other dose groups. Other commonly reported adverse events were eye irritation, abnormal sensation in the eye, ocular hyperemia, red eye, eye pain, keratitis, and headache. There were no deaths among the study subjects.

**Table 6:** Adverse events reported by ≥ 2% of subjects in any treatment group (ITT population), n (%)

**Latanoprost concentration (μg/mL)**				
	**50**	**75**	**100**	**125**
N	71	70	72	69
**Eye disorders**				
Abnormal sensation in eye	3 (4.2)	4 (5.7)	7 (9.7)	4 (5.8)
Anterior chamber disorder NOS	0 (0.0)	2 (2.9)	2 (2.8)	1 (1.4)
Conjunctival disorder NOS	2 (2.8)	1 (1.4)	0 (0.0)	0 (0.0)
Conjunctival edema	0 (0.0)	2 (2.9)	2 (2.8)	0 (0.0)
Conjunctival hyperemia	12 (16.9)	13 (18.6)	15 (20.8)	11 (15.9)
Dry eye NEC	1 (1.4)	2 (2.9)	4 (5.6)	0 (0)
Eye irritation	5 (7.0)	7 (10.0)	6 (8.3)	8 (11.6)
Eye pain	2 (2.8)	6 (8.6)	3 (4.2)	4 (5.8)
Eyelid disorder NOS	0 (0.0)	2 (2.9)	0 (0.0)	0 (0.0)
Keratitis NEC	2 (2.8)	4 (5.7)	3 (4.2)	3 (4.3)
Ocular hyperemia*	5 (7.0)	4 (5.7)	5 (6.9)	2 (2.9)
Red eye	3 (4.2)	5 (7.1)	6 (8.3)	2 (2.9)
Vision blurred	1 (1.4)	0 (0.0)	2 (2.8)	3 (4.3)
**Musculoskeletal, connective tissue and bone disorders**				
Arthralgia	1 (1.4)	0 (0.0)	2 (2.8)	1 (1.4)
**Nervous system disorders**				
Headache NOS	4 (5.6)	4 (5.7)	3 (4.2)	4 (5.8)

*ITT* intention-to-treat, *NEC* not elsewhere classified, *NOS* not otherwise specified.

*Includes conjunctival hyperemia where investigator specified it as bulbar or palpebral.

In the overall population, 78/282 (27.7%) of subjects had conjunctival hyperemia at baseline, with most reporting no worsening of the condition from baseline to week 4. Sixty subjects (15, 17, 15, and 13 subjects in the 50, 75, 100 and 125 μg/mL dose groups, respectively) experienced a worsening of conjunctival hyperemia from baseline to week 4 while 15 subjects (3, 4, 6 and 2 in the 50, 75, 100 and 125 μg/mL groups, respectively) had an improvement from baseline at week 4. One subject in the latanoprost 50 μg/mL group had severe conjunctival hyperemia at week 4 while 2 subjects (1 each in the 100 and 125 μg/mL groups) had severe conjunctival hyperemia at weeks 1 and 2, respectively. At week 4, one subject in the 75 μg/mL group had cells/flare in the anterior chamber.

Most subjects reported no specific ocular symptoms during the treatment period. One subject in the 100 μg/mL group had a severe foreign body sensation at week 2, while three subjects (1 each in the 50, 75, and 100 μg/mL groups) reported severe itching at week 2. Two subjects (1 each in the 50 and 100 μg/mL groups) developed severe eye redness at weeks 4 and 2, respectively. Over 90% of subjects showed no change from baseline in iris pigmentation or eyelashes. Weak changes in iris pigmentation (defined as slight but clearly visible) were observed in two subjects (1 each in the 100 and 125 μg/mL groups). Changes in eyelashes were observed in 11 subjects (3, 3, 4 and 1 in the 50, 75, 100 and 125 μg/mL dose groups, respectively).

## Discussion

In the primary analysis, there was no statistical difference in the change from baseline in IOP at week 4 between dose groups in the worse eye (PP population) at either 8 a.m. or 4 p.m. Additional analyses also showed no significant differences between dose groups for change from baseline in IOP at weeks 1, 2, and 3 and percent change from baseline in IOP at week 4. Patients experienced a robust IOP lowering effect of 8–10 mm Hg from baseline in all groups at all timepoints. Numerically, the IOP reduction produced by higher concentrations was at all timepoints slightly less than that produced by 50ug/ml latanoprost. The robust IOP lowering effect and the power of the study lead to the conclusion that concentrations of latanoprost higher than 50ug/ml do not produce greater IOP lowering effects. These results were in agreement with the small dose-ranging study by Alm et al. [14] \in which they found no differences in effect at concentrations of 35, 50, or 115 μg/mL at 1, 2 or 4 weeks; they did, however, report a dose effect in favor of the higher doses at 2 days. In contrast, in a recent dose-ranging study with another prostaglandin analog, bimatoprost 0.01% was found to be equivalent in efficacy to 0.03% bimatoprost, while, surprisingly, bimatoprost 0.0125% was not as effective [23].

Latanoprost was well tolerated at all concentrations, with no differences between dose groups with respect any of the adverse events, including conjunctival hyperemia. There were two reports of iris darkening and 11 reports of eyelash growth, but there was no dose association for either of these events. Both are well recognized side effects of prostaglandin analog therapy [24,25].

These results suggest that latanoprost is at the top of the dose–response curve for both IOP lowering and for hyperemia at the 50ug/ml dose. In order for latanoprost to produce IOP lowering, the drug must penetrate into the eye, be converted into the active latanoprost acid, and interact with the relevant prostaglandin receptors. The lack of increased effect of higher concentrations may indicate saturation of the penetration or conversion process rather than the saturation of the receptors. However, application of latanoprost more than once a day to increase the overall drug exposure does not increase IOP lowering [19], so saturation of the penetration or conversion mechanisms is unlikely.

In addition, the observation that both IOP and hyperemia are stable over this dose range suggests that both effects are mediated by the interaction of latanoprost with same receptor. In contrast to our finding that hyperemia does not increase as the concentration of latanoprost increases over this range, dose finding studies of bimatoprost [23] suggest that while the 0.01% concentration of bimatoprost is at the top of the dose–response curve for IOP reduction, higher doses produce additional hyperemia, suggesting that bimatoprost may interact with hyperemia-producing receptors that do not mediate IOP reduction.

## Conclusions

Robust decreases from baseline in IOP were observed in all treatment groups, with no significant differences between the marketed concentrations of latanoprost (50 μg/mL) and any of the higher concentrations (75, 100, and 125 μg/ml) of latanoprost. All concentrations of latanoprost were well tolerated, with no dose association of any adverse event. Both IOP lowering and hyperemia appear to be unrelated to dose across the range tested.

## Competing interests

The authors are employees of Pfizer Inc (DE, CT) or Pfizer Ltd (CS).

## Authors’ contributions

Individual contributions are listed below: *Study concept and design: CS,CT, DE Acquisition of data: CS Analysis and interpretation of data: CS, CT, DE Drafting of the manuscript: CTCritical revision of the manuscript for important intellectual content: CS, CT, DE Study supervision: CS.* All authors read and approved the final manuscript.

## Funding

The study was sponsored by Pharmacia Upjohn which was acquired by Pfizer in 2003.

## Pre-publication history

The pre-publication history for this paper can be accessed here:

http://www.biomedcentral.com/1471-2415/12/9/prepub
